# Early Versus Late Endotracheal Intubation in Subjects with COVID-19 Pneumonia Treated with High-Flow Oxygen: A Retrospective Observational Study

**DOI:** 10.7759/cureus.47488

**Published:** 2023-10-22

**Authors:** Rashid Nadeem, Muhannad Alheraki, Farooq Dar, Karim S Hussein, Hina Mirza, Ishma Aijazi, Moatz G ElZeiny, Neama A Awadh, Hadeel Osman, Rawan Albwidani

**Affiliations:** 1 Intensive Care Medicine, Dubai Hospital, Dubai, ARE; 2 Internal Medicine, Dubai Hospital, Dubai, ARE; 3 Cardiothoracic Surgery, Dubai Health Authority, Dubai, ARE; 4 Intensive Care Unit, Dubai Hospital, Dubai, ARE; 5 Family Medicine, Dubai Academic Health Corporation, Dubai, ARE; 6 Intensive Care Medicine, Dubai Hospital, Dubai Health Authority, Dubai, ARE

**Keywords:** high-flow oxygen, airway intubation, acute respiratory distress syndrome, pneumonia, covid-19

## Abstract

Introduction: The availability of high-flow oxygen (HFO) machines allowed patients with COVID-19 pneumonia to be comfortably treated for longer periods of time until endotracheal intubation became inevitable. Patients treated with invasive mechanical ventilation (MV) preceded by HFO treatment may continue to progress and die. Hence there is a belief in physicians that patients treated with HFO might have delayed invasive MV.

Methods: The study was conducted as a retrospective review of subjects with confirmed COVID-19 admitted to the Dubai Hospital ICU. Study variables included time to intubation, duration of HFO, and cumulative duration of tachypnea and tachycardia while on HFO usage. Early intubation was defined as within 24 hours of the start of HFO, and late intubation was defined as after seven days on HFO. Groups were compared for outcome measures; mortality and length of stay (LOS) in the ICU and hospital.

Results: Clinical outcomes of mortality and LOS in ICU and hospital were not significantly different among patients intubated early versus late. Duration of tachypnea and tachycardia was also not different comparing patients intubated early versus late.

Conclusion: There was no significant difference in clinical outcomes in patients intubated early versus late in patients treated with HFO for COVID-19 pneumonia.

## Introduction

COVID-19, caused by the SARS-CoV-2, has resulted in a global pandemic, with a significant impact on healthcare systems worldwide [1]. Mortality rates improved in the later part of the pandemic in 2021 [2-4]. In the first phase of the pandemic (2020), guidelines recommended early intubations as most patients were rapidly deteriorating [5]. Studies showed later that the use of HFO through a nasal cannula significantly decreased the need for mechanical ventilation (MV) support [6]. These patients with lung injury usually have a high respiratory drive, resulting in intense inspiratory effort causing further lung damage by several mechanisms gathered under the name “patient self-inflicted lung injury” (P-SILI) [7]. Subjects treated with HFO for prolonged periods of time may develop further lung damage affecting clinical outcomes [8]. Clinical observation suggests some patients who are treated with HFO if eventually required endotracheal invasive MV suffer bad outcomes suggesting that these patients may continue to suffer from higher work of breathing with occult signs of exhaustion.

We hypothesize that subjects who had longer periods of treatment with HFO (who were intubated late) may have worse outcomes since they suffer from gradual progressive pulmonary alveolar damage from PSILI without showing overt signs of respiratory failure. We also hypothesize that occult signs of exhaustion, longer duration of high respiratory rates, and longer duration of tachycardia may be associated with poor survival.

The primary aim was to compare the clinical outcomes in patients who were initially treated with HFO followed by early versus late endotracheal intubation. The hypothesis was that patients treated with longer periods of HFO (late intubations) had poor clinical outcomes compared to the subjects who were treated with a shorter duration of HFO (early intubation). The secondary aim was to observe if patients who exhibited a longer duration of tachypnea and tachycardia had poor clinical outcomes.

## Materials and methods

A retrospective observational chart review study was performed on all subjects with confirmed COVID-19 pneumonia admitted to the Dubai Hospital ICU between January 01, 2021, and December 31, 2021, who were treated with invasive mechanical ventilation (MV). All subjects who did not require invasive MV were excluded. Subjects <18 years of age and pregnant subjects were also excluded. Subjects with chronic lung diseases, chronic obstructive pulmonary disease, asthma, pulmonary fibrosis, coronary artery disease, or congestive heart failure were not excluded. If the subject was readmitted to the ICU, only data from index admission was included.

All decisions to intubate were made by clinicians taking care of subjects based on clinical judgment, that is, respiratory distress, use of accessory muscles, oxyhemoglobin desaturations, or hemodynamic instability.

The primary variable of interest was intubation time, early (within 96 hours of the start of HFO) and late intubation (after 96 hours). We identified a cut-off of early and late as the median time of HFO was 96 hours.

The secondary variable of interest was the cumulative duration of tachypnea (respiratory rate (RR) >30/min) and tachycardia (≥100 bpm) while on the HFO in intubated patients. Clinical outcome variables: mortality and length of stay (LOS) in ICU and hospital. 

Confounding factors affecting clinical outcomes were also recorded. The demographics recorded were as follows: age; gender; body mass index (BMI); data on comorbidities including diabetes, hypertension, coronary artery disease, renal failure, and outpatient dialysis. Inpatient clinical data on admission to ICU including fever (temperature: >38℃), tachycardia, hypotension defined as systolic blood pressure (SBP) <90 mmHg, use of oxygen (L/min), MV, pressers, and inpatient dialysis were also recorded.

Titration of oxygen therapy was based upon hospital protocol, with a gradual increase of oxygen if a subject exhibited an oxygen saturation (SpO_2_) of <90% as measured by pulse oximetry.

All pertinent data was retrieved from the Salama® electronic medical record system 2017 (Dubai Health Authority, United Arab Emirates).

Statistical analysis

Chi-square tests were performed for categorical variables. Mann-Whitney U test for continuous variables was performed when data were not normally distributed. Logistic regression was performed to assess if survival is predicted by early versus late intubation. Kaplan-Meier plots were also constructed to analyze the survival patterns of subjects with early versus late intubations.

Simple and multiple linear regressions were used to analyze data on ICU LOS and hospital LOS.

A p-value of 0.05 was considered significant. All analyses were performed with SPSS version 28 (IBM Corp., Armonk, USA).

## Results

Screening for the sample revealed 303 patients with COVID-19 pneumonia who required invasive MV. The sample included 193 subjects treated with non-invasive MV (BIPAP) instead of HFO. HFO was used for 110 patients; 67 of these were switched to BIPAP before proceeding to intubation so there were 43 subjects who were only treated with HFO followed by intubation. Twenty-one patients underwent early intubation while 22 patients underwent late intubation. Sample characteristics are described in Table 1 and Table 2. Subjects had HFO time (median of 96 hours with an interquartile range (IQR) of 139 hours). Cumulative tachypnea duration was a median of 23 hours with an IQR of 69 hours. Cumulative tachycardia duration was a median of two hours with an IQR of 15 hours.

**Table 1:** Sample characteristics stratified by early versus late intubation Data has been presented as N, %, median, and interquartile range. The p-value is considered significant (p<0.05) IQR, interquartile range

Variable	Sample (n=43), n (%)	Early intubation, N=21	Late intubation, N=22	P-value
Time duration on high flow O_2_ (h) --->	Median (IQR) 96 (139) hours	Median (IQR) 31 (32) hours	Median (IQR) 169 (184) hours	0.01
Gender (M/F)	31/12	17/4	14/8	0.31
Diabetes	26 (60.5%)	15 (78.9%)	11 (78.6%)	1.00
Hypertension	18 (41.8%)	4 (25%)	14 (77.8%)	0.01
Coronary disease	7 (16.2%)	4 (26.7%)	3 (21.4%)	1.00
Renal failure	12 (27.9%)	4 (19%)	7 (33.3%)	0.48
Outpatient dialysis	4 (9.3%)	2 (9.5%)	2 (9.1%)	1.00
Immunosuppressed	9 (20.9%)	6 (28.6%)	3 (13.6%)	0.28
Hypotension	7 (16.2%)	3 (14.4%)	4 (18.2%)	1.00
Vasopressors	28 (65.1%)	14 (66.7%)	14 (63.6%)	1.00
Inpatient dialysis	12 (27.9%)	6 (28.6%)	5 (22.7%)	0.73
Bacterial infection	26 (60.4%)	13 (61.9%)	13 (59.1%)	1.00
Steroids	42 (97.6)	21 (100%)	21 (95.5%)	1.0
Tracheostomy	8 (18.6%)	4 (19%)	4 (18.2%)	1.00
Died	23 (53.4%)	9 (42.9%)	14 (63.6%)	0.22

**Table 2:** Sample characteristics stratified by early versus late intubation (continuous variables) Data has been presented as N, median, and interquartile range. The p-value is considered significant (p<0.05). BMI, body mass index; APACHE, Acute Physiology and Chronic Health Evaluation; MV, mechanical ventilation; LOS, length of stay; HF, high flow; RR, respiratory rate

Continuous variables	Early intubation, n=21		Late intubation, n=22		P-value
	Median	IQR	Median	IQR	
Age (years)	45.5	15	55.5	20	0.009
BMI (Kg/m^2^)	29.5	4.1	29	2.7	0.889
APACHE 2 scores	55	24	52	17	0.450
Days on MV	9.5	6.3	13.5	8.8	0.694
ICU LOS	14.5	12	18.5	6.8	0.808
Hospital LOS	27	11	29.5	7	0.267
Time on HF (h)	31.5	24.5	205	254	0.001
Time duration of tachypnea RR >30 (h)	24.5	29	105	177	0.001
Time duration of tachycardia pulse >100 (h)	1.5	1.5	1	2.2	0.110

There was no significant difference between survivors versus non-survivors; nine of 21 subjects died in the early intubation group versus 14 of 22 in the late intubation group p=0.14, and there was no significant difference in LOS ICU 14 (12.5) vs. 18 (23.5) days, p=0.80, and for hospital LOS 23 (18) vs. 29.5 (17), p=0.26 (Tables 3-5).

**Table 3:** Comparison of mortality between early versus late intubation Data has been presented as N, median, and interquartile range. The p-value is considered significant (p<0.05). IQR, interquartile range

	Alive, number	Median (hours)	IQR (hours)	Died, number	Median (hours)	IQR (hours)	P-value
Early intubation	12	32	41.4	9	24	29.5	0.862
Late intubation	8	192	300	14	169	183	0.868

**Table 4:** Length of stay in ICU in patients with early versus late intubation Data is presented as N, median, and interquartile range; The p-value is considered significant if P<0.05. IQR, interquartile range

	ICU Length of stay (days)						P-value
	Alive			Died			
	Number	Median (hours)	IQR (hours)	Number	Median (hours)	IQR (hours)	
Early intubation	12	19.5	21.8	9	8	6.5	0.002
Late intubation	8	19	32	14	10.5	25	0.95

**Table 5:** Length of stay in hospital in patients with early versus late intubations Data has been presented as N, median, and interquartile range. The p-value is considered significant (p<0.05). IQR, interquartile range

	Hospital length of stay (days)						P-value
	Alive			Died			
	N	Median (hours)	IQR (hours)	N	Median (hours)	IQR (hours)	
Early intubation	12	33.5	25	9	19	15	0.004
Late intubation	8	32	36	12	27	19	0.069

Tachypnea duration was longer for patients in the late intubation group (3 (25) vs. 55 (106), p=0.01). Tachypnea duration for those alive versus those who died was not different (15.5 (76) vs. 23 (68), p=0.71).

Tachycardia duration was not different between the two groups (1 (3) vs. 4 (30.7) hours, p=0.11). Tachycardia duration was also not different between those who survived versus those who died (1.5 (4.3) vs. 2 (24), p=0.73).

Kaplan-Meier survival plots did not reveal any difference between early versus late intubation groups. Moreover, logistic regression analysis and linear regression analysis did not find any clinical variable predicting outcomes other than tracheostomy.

In summary, early versus late intubation has no significant difference in any clinical outcomes.

## Discussion

Our study did not find any difference in clinical outcomes between the early and late intubation groups. Lee et al. also documented no improved mortality with early intubation [9]. Ahmed et al. reported a better mortality rate with early endotracheal intubation [10]. A meta-analysis of 12 studies, by Papoutsi et al., concluded that the timing of intubation may not affect the mortality and morbidity of critically ill subjects with COVID-19 [11]. Our study results suggest that patients receiving HFO that failed and require early intubation (i.e., within 96 hours of starting HFO) versus late intubation after 96 hours of HFO have no difference in mortality or LOS in the ICU or hospital. Chandel et al. found similar results that the duration of failed HFO prior to invasive MV has no impact on clinical outcomes [12]. They use 48 hours to define early versus late intubations. Mortality and LOS in hospital were not different between the two groups. Kang et al. showed that failure of HFO causes delayed intubation and adverse clinical outcomes in patients with respiratory failure [13]. Their study used the definition of early intubation as 48 hours while we used 96 hours, which could explain the difference in findings. Our early intubation group might have included patients with late intubation by their criteria. They used 48 hours arbitrarily while we used our sample’s median duration, which was 96 hours, so it was not arbitrarily defined. Their sample was predominantly comprised of relatively older subjects (median age of 69) while we have predominantly younger subjects (median age 46 years). Younger patients may have larger pulmonary reserves enabling them to tolerate a longer duration of HFO without exhaustion. Another reason was our small sample size which may not have enough power to detect the difference in outcome if such exists. Kang et al. hypothesized that respiratory muscle fatigue and cardiac dysfunction in the late intubation group may lead to poor hospital outcomes, although they did not measure any pulmonary or cardiac parameters of fatigue. We recorded the cumulative time duration of tachypnea (respiratory rate: >30/min) and cumulative time duration of tachycardia (heart rate: >100) as a measure of respiratory and cardiac fatigue and found that the late intubation group suffered from long periods of tachypnea without long periods of tachycardia. Longer tachypnea duration was not found to have an association with worse clinical outcomes. The early intubation group in our study was found to have longer stays in the ICU and in the hospital in patients who survived versus those who died. This was a similar finding in the study by Kang et al. as most patients who died in the late intubation group had shorter stays; hence, they died in the ICU rather than outside the ICU.

We hypothesize that the optimal time for intubation is different for patients with different profiles. A younger patient without comorbid conditions with a better pulmonary reserve may tolerate long periods of HFO treatment in contrast to an older subject with a limited pulmonary and cardiac reserve who may not tolerate long periods of HFO. Hence the arbitrary definition of early and late may not be applicable. Instead, the objective parameter of pulmonary reserve may be more useful. This could be the duration of tachypnea, the severity of tachypnea, or the trajectory of tachypnea development and is a matter of debate. Similarly, this could be the duration of tachycardia, the severity of tachycardia, or the trajectory of tachycardia development. We measured these two clinical variables and compared the two groups and found that tachypnea duration was significantly longer in patients with the late intubation group. Tachycardia duration was similar. We believe tachypnea as a measure of pulmonary exhaustion can be a potential target to define the optimal time to intubate patients treated with HFO. 

Our study has many limitations; a small sample size precluded us from detecting if there was a difference in outcomes with statistical power. Our population comprised of young male patients without significant prior lung disease or other comorbidities. So, our finding may not be applicable to other populations although this is the message we learned from our study that the optimal time of intubation may be population-specific. Patients who failed HFO treatment but had a trial of non-invasive MV before intubation had to be excluded as we had no control over the treatment provided as the study was retrospective. If we include those our sample size and power could be improved. 

## Conclusions

Subjects who are treated with HFO seem to have no clinical difference in outcomes with early or late intubation. Patients treated with prolonged use of HFO do not appear to have an adverse impact on mortality or LOS in the ICU or hospital. The patients with late intubation exhibit prolonged tachypnea. Although prolonged tachypnea does not predict mortality. This study lacks the power to determine the association between early versus late intubation to clinical outcomes. Large prospective trials should be considered to determine the optimal time for invasive MV in patients treated with HFO.

## References

[1] (2023). WHO Coronavirus (COVID-19) Dashboard. https://covid19.who.int/.

[2] Finelli L, Gupta V, Petigara T, Yu K, Bauer KA, Puzniak LA (2021). Mortality among US patients hospitalized with SARS-CoV-2 infection in 2020. JAMA Netw Open.

[3] Roth GA, Emmons-Bell S, Alger HM (2021). Trends in patient characteristics and COVID-19 in-hospital mortality in the United States during the COVID-19 pandemic. JAMA Netw Open.

[4] Horby P, Lim WS, Emberson JR (2021). Dexamethasone in hospitalized patients with COVID-19. N Engl J Med.

[5] Brown CA II, Mosier JM, Carlson JN, Gibbs MA (2020). Pragmatic recommendations for intubating critically ill patients with suspected COVID‐19. J Am Coll Emerg Physicians Open.

[6] Ospina-Tascón GA, Calderón-Tapia LE, García AF (2021). Effect of high-flow oxygen therapy vs conventional oxygen therapy on invasive mechanical ventilation and clinical recovery in patients with severe COVID-19: a randomized clinical trial. J Am Med Assoc.

[7] Brochard L, Slutsky A, Pesenti A (2017). Mechanical ventilation to minimize progression of lung injury in acute respiratory failure. Am J Respir Crit Care Med.

[8] Carteaux G, Parfait M, Combet M, Haudebourg AF, Tuffet S, Mekontso Dessap A (2021). Patient-self inflicted lung injury: a practical review. J Clin Med.

[9] Lee YH, Choi KJ, Choi SH, Lee SY, Kim KC, Kim EJ, Lee J (2020). Clinical significance of timing of intubation in critically ill patients with COVID-19: a multi-center retrospective study. J Clin Med.

[10] Ahmad I, Jeyarajah J, Nair G, Ragbourne SC, Vowles B, Wong DJ, El-Boghdadly K (2020). A prospective, observational, cohort study of airway management of patients with COVID-19 by specialist tracheal intubation teams. Can J Anaesth.

[11] Papoutsi E, Giannakoulis VG, Xourgia E, Routsi C, Kotanidou A, Siempos II (2021). Effect of timing of intubation on clinical outcomes of critically ill patients with COVID-19: a systematic review and meta-analysis of non-randomized cohort studies. Crit Care.

[12] Chandel A, Patolia S, Brown AW (2021). High-flow nasal cannula therapy in COVID-19: using the ROX index to predict success. Respir Care.

[13] Kang BJ, Koh Y, Lim CM (2015). Failure of high-flow nasal cannula therapy may delay intubation and increase mortality. Intensive Care Med.

